# Network and Data Integration for Biomarker Signature Discovery via Network Smoothed T-Statistics

**DOI:** 10.1371/journal.pone.0073074

**Published:** 2013-09-03

**Authors:** Yupeng Cun, Holger Fröhlich

**Affiliations:** Algorithmic Bioinformatics, Bonn-Aachen International Center for IT, Bonn, Germany; Technical University of Madrid, Italy

## Abstract

Predictive, stable and interpretable gene signatures are generally seen as an important step towards a better personalized medicine. During the last decade various methods have been proposed for that purpose. However, one important obstacle for making gene signatures a standard tool in clinics is the typical low reproducibility of signatures combined with the difficulty to achieve a clear biological interpretation. For that purpose in the last years there has been a growing interest in approaches that try to integrate information from molecular interaction networks. We here propose a technique that integrates network information as well as different kinds of experimental data (here exemplified by mRNA and miRNA expression) into one classifier. This is done by smoothing t-statistics of individual genes or miRNAs over the structure of a combined protein-protein interaction (PPI) and miRNA-target gene network. A permutation test is conducted to select features in a highly consistent manner, and subsequently a Support Vector Machine (SVM) classifier is trained. Compared to several other competing methods our algorithm reveals an overall better prediction performance for early versus late disease relapse and a higher signature stability. Moreover, obtained gene lists can be clearly associated to biological knowledge, such as known disease genes and KEGG pathways. We demonstrate that our data integration strategy can improve classification performance compared to using a single data source only. Our method, called stSVM, is available in R-package netClass on CRAN (http://cran.r-project.org).

## Introduction

One of the major goals of personalized medicine is to identify reliable molecular biomarkers that predict relevant clinical characteristics for an individual patient, like disease sub-type, his/her response to a certain therapy or survival time. Prognostic and diagnostic biomarker signatures can nowadays be constructed on the basis of multiple molecular data, such as gene expression data, miRNA, methylation or copy number alterations [1].

A common approach to obtain a signature for diagnostic or prognostic purposes is to put patients into distinct groups and then construct a classifier that can discriminative patients in the training set and is able to predict well unseen patients. Frequently applied algorithms are Prediction Analysis for Microarrays (PAM) [2], Support Vector Machine Recursive Feature Elimination (SVM-RFE) [3], Random Forests [4] or statistical tests, like Significant Analysis for Microarrays (SAM) [5], combined with machine learning techniques (SVM, k-NN, linear discriminant analysis, logistic regression,…) [6], [7]. However, a commonly encountered problem is that molecular signatures are often not reproducible in the sense that inclusion or exclusion of a few patients can lead to quite different sets of selected features. Moreover, these sets are often difficult to interpret in a biological way [8]. Both issues currently prevent molecular signatures to become a standard tool in clinical practice [9]. For that reason, various network based approaches have been proposed to integrate prior knowledge on canonical pathways, Gene Ontology (GO) annotation or protein-protein interactions into feature selection algorithms [10]–[17]. A recent review on such approaches can be found in [18]. The general hope of these approaches is that biological knowledge can lead to better interpretable and more stable signatures. Whether network based classification methods automatically also lead to higher prediction accuracies is still a matter of debate [19], [20].

Another line of research focuses on the integration of different entities of experimental data for the same patient, e.g. mRNA and miRNA expression [21]–[24]. The increasing amount of different kinds of molecular data from the same patient, for instance within the TCGA database (www.cancergenome.nih.gov), now opens the door to a broader disease understanding [25]–[27]. Moreover, the integration of data capturing different molecular mechanisms could also lead to improved molecular signatures.

In this paper we propose a filter based feature selection approach, which integrates network information by smoothing gene-wise t-statistics over the graph structure using a random walk kernel. Our approach allows for a straight forward integration of different data entities, like mRNA and miRNA expression. Comparisons of our smoothed t-statistic SVM (stSVM) with several competing approaches on a breast cancer, two prostate cancer and an ovarian cancer dataset demonstrate a favorable prediction performance of early versus late relapse and a high signature stability. Moreover, obtained gene lists are highly enriched with known disease genes and KEGG pathways.

## Materials and Methods

### Datasets

We retrieved one breast cancer [28], two prostate cancer [29], [30] and one ovarian cancer [26] dataset from different data repositories. The breast cancer [28] and one of the prostate cancer datasets [29] were measured on Affymetrix HGU133 microarrays. The second prostate cancer dataset (MSKCC, [30]) and the ovarian cancer dataset (TCGA, [26]) were measured on Affymetrix HuEx 1.0 ST microarrays. The breast and first prostate cancer dataset were normalized via FARMS [31]. The ovarian cancer and MSKCC datasets were downloaded as ready normalized and gene-wise aggregated data from the TCGA and MSKCC homepage, respectively. As clinical end points we considered metastasis free (breast and prostate cancer) and relapse free (ovarian cancer) survival time after initial clinical treatment. For ovarian cancer only tumors with stages IIA - IV and grades G2 and G3 were considered, which after resection revealed at most 10 mm residual cancer tissue and responded completely to initial chemotherapy.

Survival time information was dichotomized into two classes according whether or not patients suffered from a reported relapse/metastasis event within 5 years (breast, prostate dataset 1), 3 years (MSKCC prostate cancer dataset) and 1 year (ovarian), respectively. Patients with a survival time shorter than 5/3/1 year(s) without any reported event were not considered and removed from our datasets. This was done, because these patients can neither reliably be put into the early nor into the late relapse class. A summary of our datasets can be found in Table 1.

**Table 1:** Overview about employed datasets.

ID/source	patients	cancer type	classification	positive class
GSE4922	228	breast	metastasis free survival >5 y	69
TCGA	135	ovarian	relapse free survival >1 y	35
GSE21032(MSKCC)	79	prostate	relapse free survival >3 y	29
GSE25136	79	prostate	recurrent vs. non-recurrent	40

### Network Information

#### Protein-Protein Interactions (PPI)

A comprehensive protein interaction network was compiled from the Pathway Commons database [32], which was downloaded in tab-delimited format (September 2012). All SIF interactions INTERACTS_WITH and STATE_CHANGE were taken into account (http://www.pathwaycommons.org/pc/sif_interaction_rules.do) and self loops removed, resulting in a large network with 11,361 nodes and 610,185 edges. Nodes in this network were identified with Entrez gene IDs. Expression values for probesets on the microarray that mapped to the same gene in the network were averaged. In order to consider genes with available probesets on the array but no corresponding network information we added for all these genes unconnected nodes to our initial network, resulting in 12,611 nodes for breast and the Sun et al. prostate cancer dataset; 11,356 nodes for ovarian cancer and 11,322 nodes for the MSKCC prostate cancer dataset. The reason for these differences is that not all dataset contain the same number of mappable transcripts.

#### KEGG pathways

As an alternative network information we computed a merger of all non-metabolic KEGG pathways [33]. For retrieval and merger of KEGG pathways, we employed the R-package KEGGgraph [34]. Only gene-gene interactions were considered, which resulted in an initial network with 3,087 nodes and 17,518 edges. As before this initial network was extended to contain all genes available on the array, resulting in an overall network with the same number of nodes as described above for the PPI network but a different number of edges.

#### miRNA-Target gene network

In addition to PPI or KEGG pathway information we optionally included predicted miRNA-target gene interactions. Target predictions were obtained from the MicroCosm database (version 5) [35] (FDR cutoff 1%). This increased the number of edges in the PPI network to 11,892 nodes for MSKCC’s prostate cancer and 11,839 nodes for ovarian cancer.

### Prediction Performance, Signature Stability and Biological Interpretability

In order to assess the prediction performance of all tested methods we performed a 10 times repeated 10-fold cross-validation on each dataset. That means the whole data was randomly split into 10 fold, and each fold sequentially left out once for testing, while the rest of the data was used for training and optimizing the classifier (including selection of relevant genes, hyper-parameter tuning, standardization of expression values for each gene to mean 0 and standard deviation 1, etc.). The whole process was repeated 10 times. It should be noted extra that also standardization of gene expression data was only done on each training set separately and the corresponding scaling parameters then applied to the test data.

The area under receiver operator characteristic curve (AUC) was used to measure the prediction accuracy via the R-package ROCR [36]. To assess the stability of gene selection, we computed the selection frequency of each gene within the 10 times repeated 10-fold cross-validation procedure. That means a particular gene could be selected at most 100 times In order to summarize the selection frequencies for all genes we defined a so-called stability index (SI) as
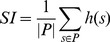
(1)where 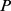 is the set of selected genes that had been selected at least once and 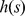 is the actual number of times that 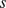 was selected. SI represents a weighted histogram count of selection frequencies. Obviously, the larger SI the more stable the algorithm is. In the optimal case 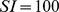. The 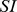 has to be seen together with the size of gene signature, because trivially a classifier selecting all genes would always achieve 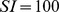.

In order to check in how far signatures obtained by training the classifier on the whole dataset could be related to existing biological knowledge, we looked for enrichment of disease related genes via the tool FunDO [37] (hypergeometric test; multiple testing correction: Bonferroni’s method). Moreover, we calculated the enrichment with KEGG pathways [33] via a hyper-geometric test.

### Network Smoothed T-Statistic SVMs (stSVMs)

#### Network Smoothed T-Statistics

Given a simple, undirected graph 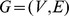 with adjacency matrix 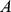 the graph Laplacian 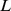 is defined as 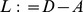, where 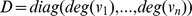 is a diagonal matrix of node degrees for nodes 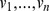 [38]. The graph Laplacian can be viewed as a discrete approximation of the negative Laplace operator for functions.

One way of characterizing the degree of relatedness of two nodes (e.g. proteins) 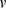 and 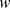 in a graph (e.g. a PPI network) can be obtained via the notion of random walks. The 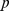-step random walk kernel is one particular similarity measure, which can be derived from this notion [39] and is defined as:
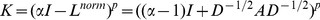
(2)


Here 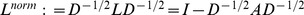 is the normalized graph Laplacian matrix, 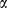 is constant, and 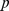 is the number of random walk steps (here: 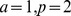). The 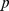-step random walk kernel gives rise to a symmetric, positive semi-definite similarity matrix between network nodes, capturing their degree of topological relatedness. The advantage compared to shortest path distance based measures is that alternative routes between pairs of nodes are considered. That means, if 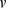 and 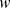 are connected via many alternative paths of the same length this marks a higher similarity than if there exists only one such path.

Suppose for each network gene we assess its differential expression on the training dataset via a t-test. This results in an absolute t-statistic 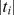 for network node 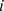. We summarize the 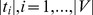 into a vector 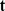 and consider the score vector
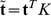
(3)


**Figure 1:**
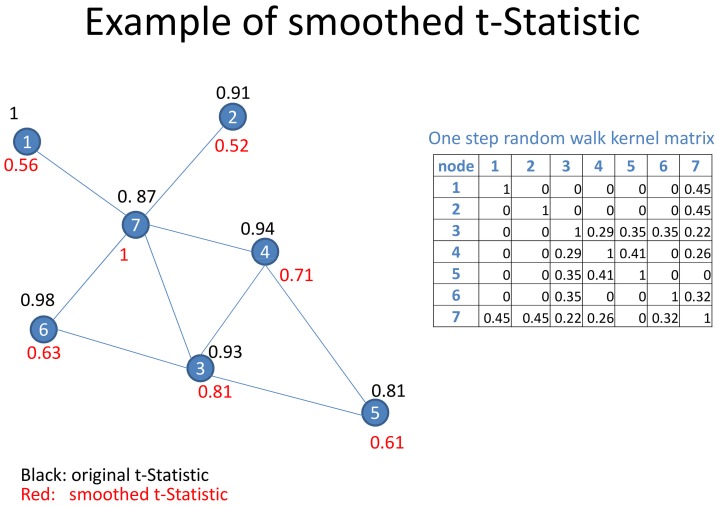
Toy example to demonstrate the network smoothed t-statistic.

Please note that 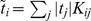. Hence, 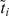 is a network smoothed version of 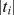 (Figure 1), but does not follow a t-distribution any more. We thus conduct a permutation test (here: 1000 times) to obtain a p-value for each gene. For reasons of computation time we restrict this to the 10% genes, which are highest ranked according to the network smoothed t-score (Eq. 3). Multiple testing correction is then performed using the FDR approach by [40].

It is worth mentioning that the smoothing of absolute t-statistics particularly affects nodes with a high number of interaction partners. On one hand our procedure aggregates the scores of neighboring nodes to increase the score for these central proteins. On the other hand there is also a reverse effect, which increases the relevance of proteins in close proximity to hubs.

#### SVM training

We only select genes with FDR <5%. Subsequently a Support Vector Machine (SVM) is trained using the optimal parameter 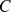 from 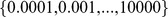. To evaluate each candidate parameter 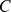 we here used the span rule, which provides a theoretical upper bound for the leave-one-out cross-validation error, but can be computed much more efficiently for datasets with few samples [41]. It has been demonstrated theoreticaly as well as empircally that the span-rule provides an excellent mechanism for parameter selection in SVMs [41]. An implementation of this procedure can be found in R-packages pathClass [42] and netClass, which is a supplement to this paper.

#### Integration of different experimental data

Besides network information our approach allows for a straight forward integrating on of different experimental data, e.g. mRNA and miRNA expression, into one classifier. This can be achieved by extending adjacency matrix 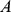 to miRNA-mRNA interactions and vector 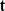 to absolute t-statistics for miRNAs. Accordingly, network smoothing is now performed over protein-protein as well as miRNA-target gene interactions.

## Results

### stSVM Shows Overall Best Prediction Performance

We initial considered our proposed stSVM method using only gene expression data and PPI network information. We compared the prediction performance to a number of competing methods, namely PAM [2], a SVM trained with significant differentially expressed genes (FDR cutoff 5%) selected by SAM [5] (**sgSVM**), average gene expression of KEGG pathways (**aepSVM** [10]), pathway activity classification (**PAC** [13]), reweighted recursive feature elimination (**RRFE** [17]) and the **netRank** algorithm [43]. NetRank, similar to RRFE, uses a modification of Google’s PageRank method to rank genes according to both, expression and network centrality [44]. The optimal number of selected genes in both cases was determined via the span-rule inside the cross-validation procedure [41].

For stSVM, netRank and RRFE, the same large PPI network was used as biological background information. The aepSVM and PAC methods use KEGG pathways. PAC relies on a so-called activity score, which is calculated per individual pathway and then taken as as a feature for classification purposes. For aepSVM we first conducted a global test [45] to select pathways being significantly associated with the class label (FDR cutoff 1%) on the training data and then calculated the mean expression of each selected pathway as a feature for SVM based classification. The prediction of all methods was assessed via a 10 times repeated 10-fold cross-validation procedure, as described in the Materials and Methods part of this paper.

**Figure 2:**
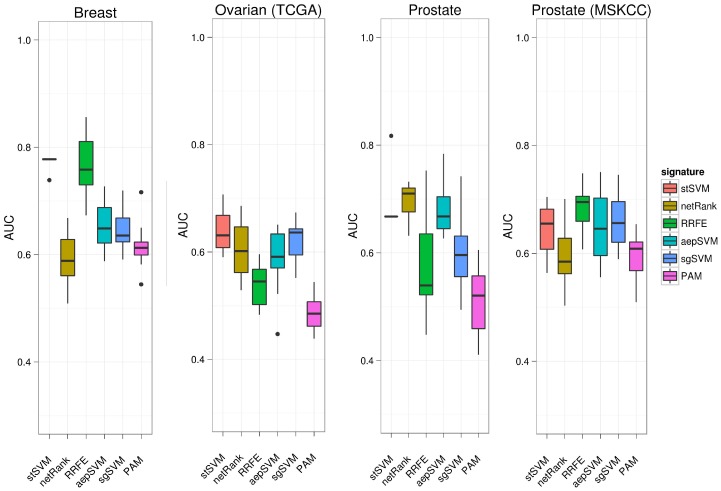
Prediction performance of stSVM in comparison to other methods in terms of area under ROC curve (AUC). Breast = GSE11121, Ovarian (TCGA) = GSE25136, Prostate = GSE25136, Prostate (MSKCC) = GSE21032.

Generally we observed a large variability of prediction performances of most tested algorithms across different datasets, which is in agreement with our previous observations [19]. However, our proposed stSVM approach showed on all of our four gene expression datasets a consistently high prediction performance with respect to the area under ROC curve (AUC, Figure 2) and significantly outperformed several competing methods (Tables S5, S6, S7, S8). Notably on two datasets (breast, prostate dataset 1) the AUC was extremely stable and showed only a very small variance across the cross-validation procedure.

In order to get a more objective and comprehensive view we conducted a ranking of all methods in each dataset according to the median cross-validated AUC value. We then calculated a consensus ranking using Kendall’s 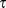 distance method [46] (Table 2). This confirmed our impression that stSVM was the overall best performing method. Interestingly enough, sgSVM was ranked second highest here, which is in agreement with our earlier finding that network based approaches do not consistently outperform classical ones [19].

**Table 2:** Ranking of different algorithms with respect to the median AUC in a 10 times repeated 10-fold cross-validation procedure.

	breast	ovarian	prostate	prostate MSKCC	consensus
**stSVM**	1	2	3	3	1
**netRank**	6	3	1	6	4
**RRFE**	2	5	5	1	3
**aepSVM**	3	4	2	4	5
**sgSVM**	4	1	4	2	2
**PAM**	5	6	6	5	6

### stSVM Yields Highly Stable Classification

We investigated the stability of signatures obtained during the 10 times repeated 10-fold cross-validation procedure using the concept of the stability index (Eq. 1), showing for stSVM an extremely robust behavior (Figure S1). Most of the signature probesets were selected consistently during the cross-validation procedure. Interestingly enough, at the same time the number of selected probesets was comparably high for stSVM, which may be attributed to the fact that the network smoothing enforces the selection of correlated genes. Tables S1, S2, S3, S4 show 10 consistently selected genes in each dataset. As expected these genes typically reveal a high node degree in the PPI network. Many of these hub genes are well known to play a role in the disease pathology, e.g. BRCA1 for all tumors [47]–[49] and AR for prostate cancer [50]. Other disease related and consistently selected genes include p53 (all datasets), EGFR (breast and prostate cancer [51], [52]), RB1 (breast and ovarian tumors [53]–[55]) and EP300 (prostate cancer [56]).

### stSVM Shows Clear Association to Biological Knowledge

**Figure 3:**
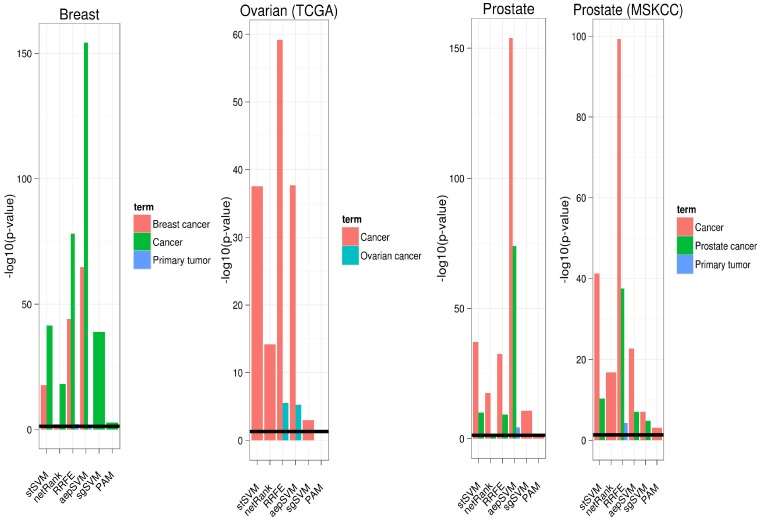
Enrichment of signatures with disease related genes. The y-axis shows -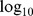 p-values computed via a hypergeometric test (Bonferroni correction for multiple testing). Black horizontal line = 5% significance cutoff.

In order to test the association with existing biological knowledge more systematically we trained each of our tested methods on complete datasets and subsequently tested the resulting signatures (Tables S9, S10, S11, S12 for stSVM, Tables S13 and S14 for stSVM(mi-mRNA) ) for enrichment of disease related genes and KEGG pathways (Figures 3, S2). For testing the association with disease related genes we used the FunDO tool [37], which is based on a hyper-geometric test.

Our analysis revealed a high enrichment of signatures obtained via stSVM to known disease genes on all datasets. The enrichment was always higher than for non-network based methods (sgSVM, PAM) as well as for signatures obtained via the netRank algorithm. The latter might be attributed to the fact that netRank typically selects only very few genes, which thus could cause a loss of statistical power for enrichment analysis.

Besides disease related genes we also found a high enrichment of stSVM derived signatures for several KEGG pathways in all datasets (Figure S2). Examples were *Pathways in cancer* (prostate, breast cancer), *Prostate Cancer* (both prostate cancer datasets), *Wnt signaling, MAPK signaling* and *ERBB signaling*. The latter three were significant in breast and prostate cancer and are known to play a role in the respective disease pathologies [57]–[63]. In ovarian cancer we particularly detected a high enrichment of several metabolic pathways, such as *Fatty acid metabolism*. This fits to the fact that adipocytes were recently found to promote rapid tumor growth in ovarian tumors [64]. The significance of enrichment for KEGG pathways was generally higher for stSVM than for all other methods.

Taken together stSVM derived signatures showed a clear association to existing biological knowledge, which eases their biological understanding.

### Influence of Network Structure

We asked the question, in how far the observed good prediction performance of stSVM was dependent on the incorporated network structure. We hence re-ran our cross-validation procedure with a different network structure, which was compiled from a merger of all non-metabolic KEGG pathways (see Materials and Methods). It is worthwhile to mention that both networks contained the same number of nodes, but different number of edges. The KEGG derived network was much sparser then the previously used PPI network.

We observed that our original PPI network in all but one case (ovarian cancer dataset) yielded significantly higher AUCs, which highlights the principle influence of the network structure (Figure S3). We can only speculate why on the ovarian cancer dataset the KEGG based network appeared to work at least as good as the PPI network. Principally KEGG pathways capture different biological aspects (canonical pathways) than large scale protein-protein interaction networks. It may be due to the nature of the disease that KEGG pathways reflect better the relevant biology for ovarian cancer than for breast and prostate tumors.

### stSVM Allows for mRNA and miRNA Data Integration

Our stSVM method allows for a straight forward integration of different types of experimental data on network level (see Materials and Methods). We here exemplify this property by using gene expression together with miRNA expression data for the TCGA ovarian cancer and for the MSKCC prostate cancer datasets. Correspondingly network information now consisted of a combined PPI and miRNA-target gene network. We call the corresponding variant of our method **stSVM(mi-mRNA)**. We compared stSVM(mi-mRNA) to the graph fusion approach by Gade et al. [21] (**GraphFusion**). In their original paper Gade et al. used CoxBoost [15] to make survival risk prediction. In our classification based framework we replaced CoxBoost by the related PathBoost algorithm [15].

Moreover, we compared stSVM(mi-mRNA) to sgSVM trained on mRNA data only, on miRNA data only and to a meta-classifier, which combines classification outputs from the mRNA and miRNA sgSVM classifiers into one consensus classifier (**sgSVM(meta)**). This was done as follows: The sgSVM method was separately trained on both datasets to yield a linear SVM classifier using significant differentially expressed genes and miRNAs, respectively. Each of these SVM classifiers yields a ranking (not classification) function of the form
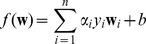
where 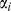 are the fitted Lagrangian multipliers, 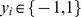 the class labels and 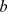 the intercept [65]. Note that the corresponding classification function can be obtained by taking the sign of 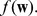 Let 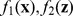 be the SVM ranking functions for mRNA profile 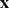 and miRNA profile 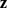, respectively. Then both rankings can be combined into a meta-classifier by fitting a logistic regression function



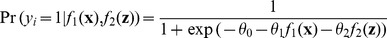
where 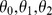 are parameters, which can be fitted to the data.

**Figure 4:**
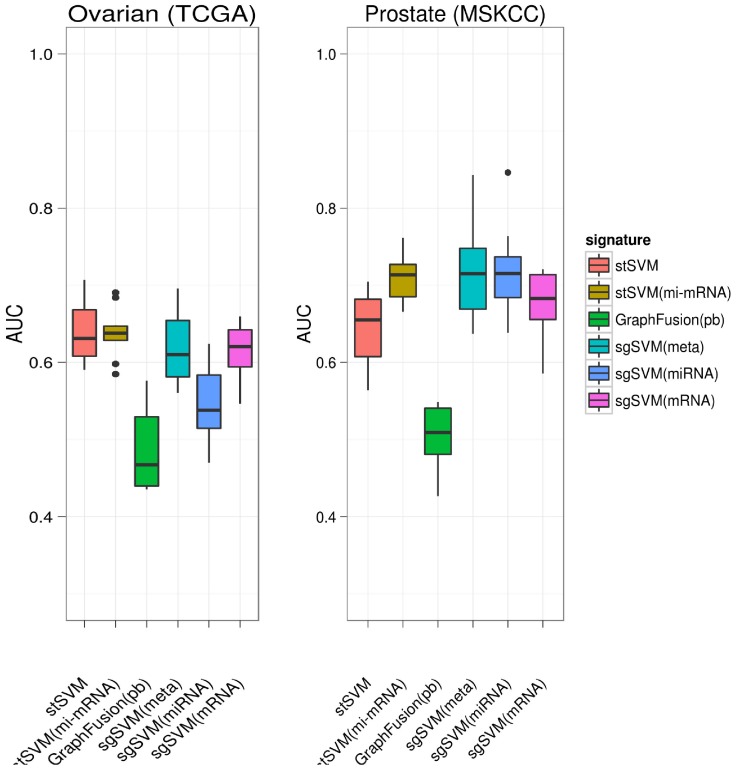
Prediction performance of stSVM on integrated gene and miRNA expression data compared to other approaches.

The comparison of our stSVM(mi-mRNA) approach to the graph fusion algorithm as well as to the above described meta-classifier approach (**sgSVM(meta)**) revealed a superior performance of our method. GraphFusion was outperformed with large margin (Figure 4), and the gain compared to sgSVM(meta) was still weakly/moderately significant (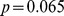 for ovarian and 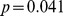 for prostate cancer; Wilcoxon signed rank test). In that context it was interesting that only on the prostate cancer dataset a significant improvement by integration of mRNA and miRNA data could be observed at all: The comparison of stSVM(meta) versus stSVM yielded a p-value of 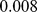 (Wilcoxon signed rank test). On the ovarian cancer dataset miRNA expression data did not appear to contribute any useful classification information. This is also highlighted by the weak performance of the sgSVM classifier trained only on miRNA expression data (**sgSVM(miRNA)**).

### Consistently Selected Features Form Disease Related Network Modules

Taking the set of genes and miRNAs, which were consistently selected by stSVM in the above investigated ovarian and MSKCC prostate cancer datasets, we asked the question, whether these features were connected to each other on network level, indicating that stSVM preferentially selected network connected genes and miRNAs.

To answer this question we looked for the largest sub-network that was purely formed by consistently selected features. In case of the ovarian cancer dataset we found 368 genes and 50 miRNAs out of 377 genes and 235 miRNAs to form such a network module. In case of the MSKCC prostate cancer dataset 384 genes and 96 miRNAs out of 386 genes and 254 miRNAs were inside one network module. This demonstrates that stSVM preferentially selected features, which were connected to each other on network level. The fraction of consistently selected genes that were inside one network module was, however, higher than the corresponding fraction of miRNAs. The reason could be that differential expression of a miRNA does not automatically imply that its target genes are also differentially expressed. Consequently miRNA markers do not always (but still in a significant proportion – see prostate cancer dataset) cluster together with gene markers on network level.

**Figure 5:**
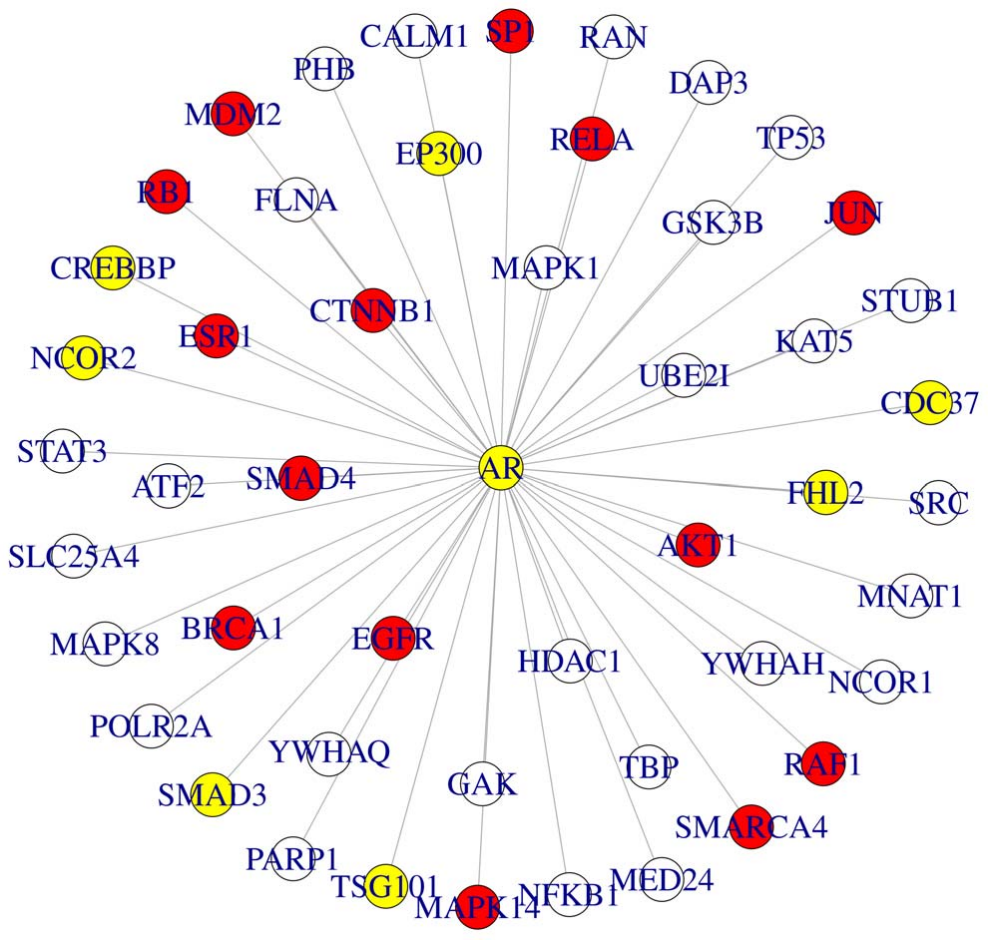
Sub-graph of disease related module identified by stSVM (MSKCC prostate cancer). The shown sub-graph consists of consistently selected genes in the interactome of the AR. For better visualization edges between neighbors of the AR are omitted. Red: cancer related genes; yellow: prostate cancer related genes.

**Figure 6:**
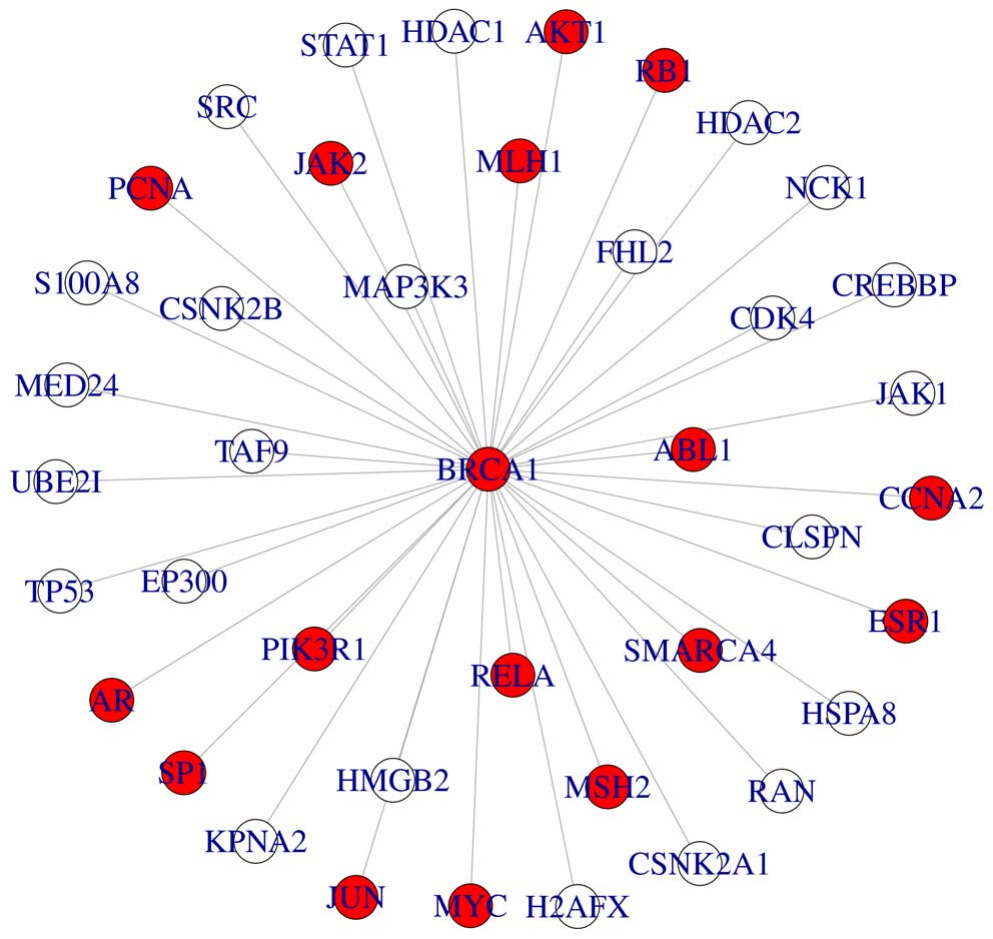
Sub-network of disease related module identified by stSVM (ovarian cancer). The shown sub-graph consists of consistently selected genes in the interactome of the BRCA1. For better visualization edges between neighbors of the BRCA1 are omitted. Red: cancer related genes.

For both, ovarian and prostate cancer, network modules were highly enriched for known disease genes (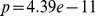 for *prostate cancer* in MSKCC prostate cancer case, 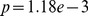 for *ovarian cancer* in ovarian cancer case) according to FunDO. Figure 5 and Figure 6 visualize sub-networks of these modules centered at the AR (MSKCC prostate cancer) and BRCA1 (ovarian cancer), respectively.

## Discussion and Conclusion

In this article we proposed network smoothed t-statistics as a method to integrate network information as well as different types of experimental data into one classifiers for biomarker signature discovery. Our method smoothed a widely used marginal statistic (the t-statistic) for differential expression over the graph structure of a biological network using random walk kernels. Our approach has on the technical level certain similarities with kernel based ranking methods for gene prioritization, which have been proposed e.g. by Moreau and co-workers to predict putative disease causing genes in genetic disorders [66]–[68]. Note, that this is a rather different problem than finding prognostic biomarker signatures.

We showed that our approach overall leads to a highly predictive, stable and biologically interpretable classifier. We exemplified the straight forward integration of different types of experimental data here by building joint classifiers of gene and miRNA expression data. Other kinds of data (e.g. methylation, copy number variations) could principally be integrated in a similar manner. This is, however, not necessarily straight forward and thus subject to future research.

Taken together we think that our method is a step towards the challenging goal to build integrative classification models, which not only make use of biological background information, but also allow to combine various kinds of molecular data in order to make accurate predictions for an individual patient. In the light of the TCGA project and other large scale efforts the time is now ripe to move into this direction.

## Supporting Information

Figure S1 **Stability index and signature sizes within the 10 times repeated 10-fold CV procedure.** A) stability index according to Eq. (1) in main document,B) number of selected probesets.
(TIF)

Figure S2 **Enrichment of signatures with KEGG pathways: Depicted is a heatmap of the -log p-value for the 10 most significant pathways.**
(TIF)

Figure S3 **Classification performance of stSVM using two different sources of network information.**
(TIF)

Table S1 **10 consistently selected genes in ovarian cancer dataset.**
(XLS)

Table S2 **10 consistently selected genes in breast cancer dataset.**
(XLS)

Table S3 **10 consistently selected genes in prostate cancer dataset (GSE25136).**
(XLS)

Table S4 **10 consistently selected genes in prostate cancer dataset (MSKCC).**
(XLS)

Table S5 **False discovery rates resulting from pairwise Wilcoxon signed rank tests to compare AUC values for different classification algorithms: breast cancer dataset.**
(XLS)

Table S6 **False discovery rates resulting from pairwise Wilcoxon signed rank tests to compare AUC values for different classification algorithms: overian cancer dataset.**
(XLS)

Table S7 **False discovery rates resulting from pairwise Wilcoxon signed rank tests to compare AUC values for different classification algorithms: prostate cancer dataset (GSE25136).**
(XLS)

Table S8 **False discovery rates resulting from pairwise Wilcoxon signed rank tests to compare AUC values for different classification algorithms: prostate cancer dataset (MSKCC).**
(XLS)

Table S9 **Final signatures obtained by stSVM in breast cancer dataset.**
(XLS)

Table S10 **Final signatures obtained by stSVM in ovarian cancer dataset.**
(XLS)

Table S11 **Final signatures obtained by stSVM in prostate cancer dataset (GSE25136).**
(XLS)

Table S12 **Final signatures obtained by stSVM in prostate cancer dataset (MSKCC).**
(XLS)

Table S13 **Final signatures obtained by stSVM(mi-mRNA) in ovarian cancer dataset.**
(XLS)

Table S14 **Final signatures obtained by stSVM(mi-mRNA) in in prostate cancer dataset (MSKCC).**
(XLS)
