# Supplementary figures and images for: Network and Data Integration for Biomarker Signature Discovery via Network Smoothed T-Statistics
Source: PLoS One. 2013 Sep 3;8(9):e73074. doi: 10.1371/journal.pone.0073074 (PMC3760887; doi:10.1371/journal.pone.0073074)

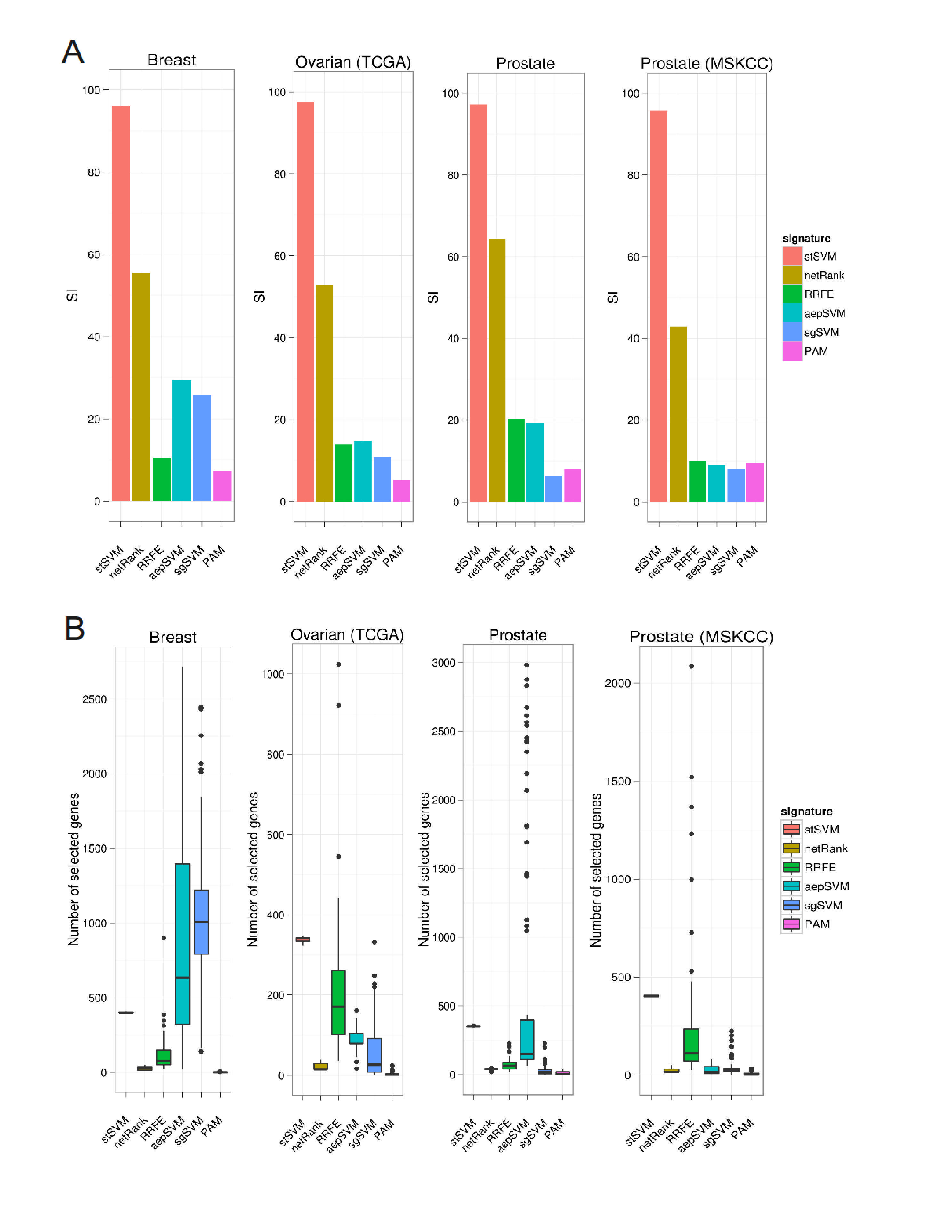

Supplement: Figure S1 — Stability index and signature sizes within the 10 times repeated 10-fold CV procedure. A) stability index according to Eq. (1) in main document,B) number of selected probesets. (TIF) [file pone.0073074.s001.tif]

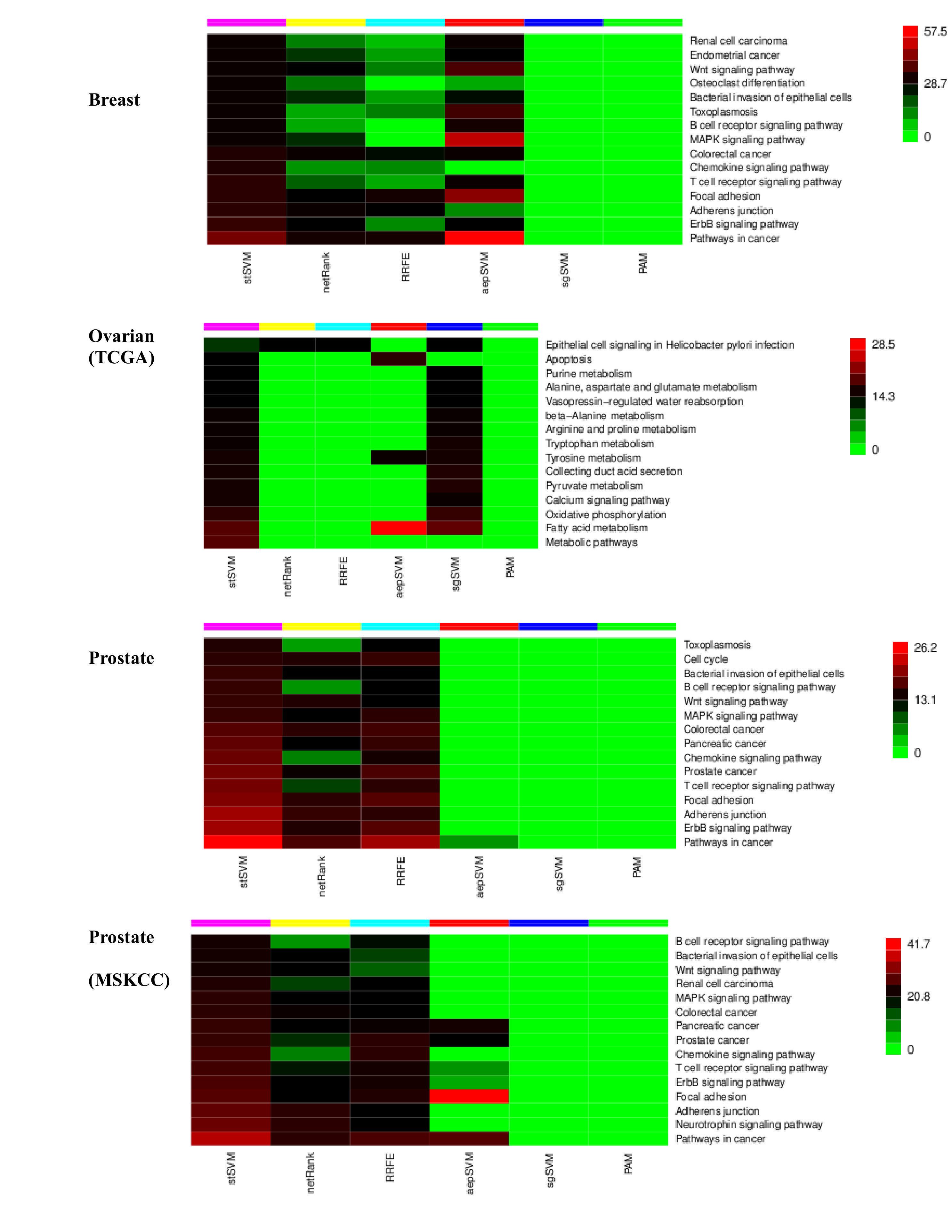

Supplement: Figure S2 — Enrichment of signatures with KEGG pathways: Depicted is a heatmap of the -log p-value for the 10 most significant pathways. (TIF) [file pone.0073074.s002.tif]

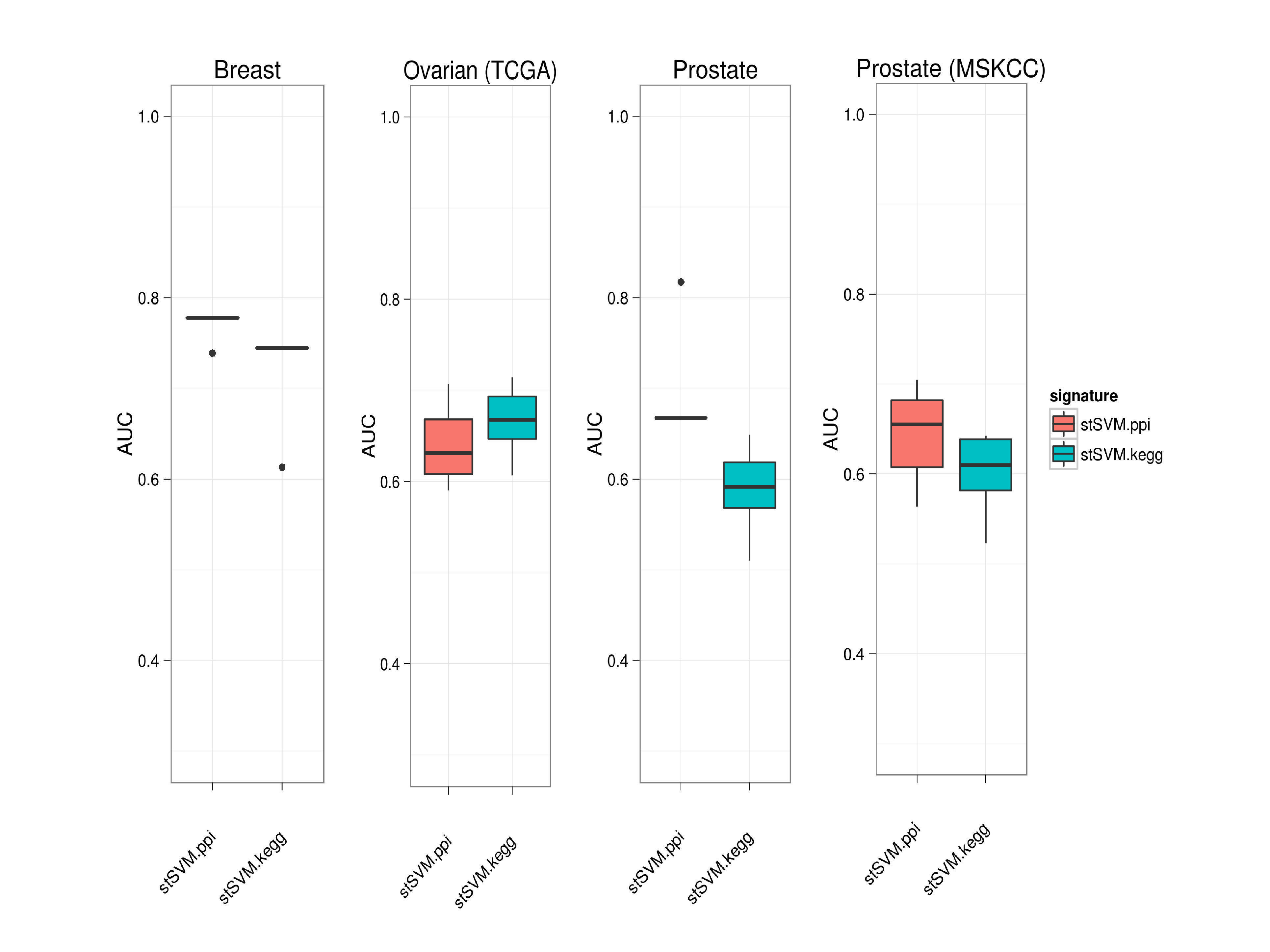

Supplement: Figure S3 — Classification performance of stSVM using two different sources of network information. (TIF) [file pone.0073074.s003.tif]
